# Peptidome analysis of umbilical cord mesenchymal stem cell **(**hUC-MSC) conditioned medium from preterm and term infants

**DOI:** 10.1186/s13287-020-01931-0

**Published:** 2020-09-23

**Authors:** Yu Wang, Lin Zhang, Yun Wu, Rongping Zhu, Yan Wang, Yan Cao, Wei Long, Chenbo Ji, Huaiyan Wang, Lianghui You

**Affiliations:** 1 Department of Neonatology, Changzhou Maternity and Child Health Care Hospital of Nanjing Medical University, Changzhou, 213000 China; 2 Nanjing Maternity and Child Health Care Institute, Women’s Hospital of Nanjing Medical University (Nanjing Maternity and Child Health Care Hospital), Nanjing, 210004 China; 3 Department of Ultrasound, Nanjing Maternity and Child Health Care Institute, Women’s Hospital of Nanjing Medical University (Nanjing Maternity and Child Health Care Hospital), Nanjing, 210004 China; 4 Department of Obstetrics, Nanjing Maternity and Child Health Care Institute, Women’s Hospital of Nanjing Medical University (Nanjing Maternity and Child Health Care Hospital), Nanjing, 210004 China

**Keywords:** hUC-MSCs, Peptidomics, LC-MS/MS, TMT labeling, Infant diseases

## Abstract

**Background:**

The therapeutic role of mesenchymal stem cells (MSCs) has been widely confirmed in several animal models of premature infant diseases. Micromolecule peptides have shown promise for the treatment of premature infant diseases. However, the potential role of peptides secreted from MSCs has not been studied. The purpose of this study is to help to broaden the knowledge of the hUC-MSC secretome at the peptide level through peptidomic profile analysis.

**Methods:**

We used tandem mass tag (TMT) labeling technology followed by tandem mass spectrometry to compare the peptidomic profile of preterm and term umbilical cord MSC (hUC-MSC) conditioned medium (CM). Gene Ontology (GO) enrichment analysis and ingenuity pathway analysis (IPA) were conducted to explore the differentially expressed peptides by predicting the functions of their precursor proteins. To evaluate the effect of candidate peptides on human lung epithelial cells stimulated by hydrogen peroxide (H_2_O_2_), quantitative real-time PCR (qRT-PCR), western blot analysis, and enzyme-linked immunosorbent assay (ELISA) were, respectively, adopted to detect inflammatory cytokines (TNF-α, IL-1β, and IL-6) expression levels at the mRNA and protein levels.

**Results:**

A total of 131 peptides derived from 106 precursor proteins were differentially expressed in the preterm hUC-MSC CM compared with the term group, comprising 37 upregulated peptides and 94 downregulated peptides. Bioinformatics analysis showed that these differentially expressed peptides may be associated with developmental disorders, inflammatory response, and organismal injury. We also found that peptides ^7118^TGAKIKLVGT^7127^ derived from MUC19 and ^508^AAAAGPANVH^517^ derived from SIX5 reduced the expression levels of TNF-α, IL-1β, and IL-6 in H_2_O_2_-treated human lung epithelial cells.

**Conclusions:**

In summary, this study provides further secretomics information on hUC-MSCs and provides a series of peptides that might have antiinflammatory effects on pulmonary epithelial cells and contribute to the prevention and treatment of respiratory diseases in premature infants.

## Introduction

The incidence of preterm birth has increased over the past 20 years in most countries [1, 2]. Despite recent advances in perinatal medicine, severe diseases related to premature birth, including periventricular leukomalacia (PVL), bronchopulmonary dysplasia (BPD), necrotizing enterocolitis (NEC), and retinopathy of prematurity (ROP), remain major causes of mortality and morbidity, which represent a heavy burden for families and society [3]. Therefore, it is an urgent and significant task to develop new safe and effective treatments to improve the prognosis of these diseases in premature infants.

In the past several decades, the development of mesenchymal stem cell (MSC) therapy and its continuous advancement have gained extensive attention. MSCs are multipotent progenitor cells, which can be raised from different tissues, for instance, adipose tissue, umbilical cord, and bone marrow [4, 5]. Human umbilical cord MSCs (hUC-MSCs) are easily accessible and can be harvested from donors without risks or damage [6]. Additionally, the therapeutic application of MSCs is not limited by the aging-like nature of adult tissues such as bone marrow and adipose tissue [7, 8]. Mechanically, MSCs function in vivo via direct differentiation or paracrine action. The therapeutic potential of MSC engraftment has been proved in premature infant diseases, and early clinical trials in preterm neonates with BPD (NCT01297205 [9], NCT01632475 [10]) and severe intraventricular hemorrhage (NCT02274428 [11]) have been conducted. A myriad of bioactive factors are readily available in the conditioned medium (CM) of MSCs and the medium can mediate multiple known functions of MSCs, such as angiogenesis, anti-fibrosis, and antiinflammatory effects [12]. Extracellular vesicles such as exosomes have been isolated from CM, and they have been shown to contain microRNAs and proteins, which partially mediated the effects of MSC [13–15]. Many studies have established that the secretome from MSCs can reduce organ damage in animal models of PVL, BPD, NEC, and ROP [16–20]. Other studies have also shown that soluble factors such as heme oxygenase-1 (HO-1) and erythropoietin (EPO) may be principally responsible for the ability of MSC-CM to ameliorate inflammation, angiogenesis, fibrosis, and so on [21, 22]. More types of MSC secreted factors and regulatory mechanisms still need to be established.

Peptides, a type of compound with two or more amino acids connected by peptide bonds, have been shown to play important roles in the treatment of diseases. Glucagon-like peptide-1 (GLP-1), a well-known peptide hormone secreted from the L cells of the duodenum, colon, terminal ileum, and rectal mucosa, has been used in the clinical treatment of type 2 diabetes [23]. Extrinsic calcitonin gene-related peptide (CGRP) could suppress apoptosis, oxidative stress, and ROS production in hyperoxia-induced alveolar epithelium type II (AECII) cells [24]. WKYMVm hexapeptide could attenuate hyperoxia-induced lung injuries in newborn mice [25]. Additionally, peptides from human milk such as PDC213, β-casein 197, and Casein201 exhibited obvious antimicrobial effects on the common pathogenic bacterial species *S. aureus* and *Y. enterocolitica* in neonatal intensive care units [26–28]. These studies indicated that peptides may hold great promise for the treatment of premature infant diseases. However, the secreted peptidomic profile from hUC-MSCs has not been fully characterized.

In the present study, we compared the secreted peptides from preterm and term hUC-MSCs using the tandem mass tag (TMT) labeling method with liquid chromatography (LC)-tandem mass spectrometry (MS/MS) analysis. Moreover, using ingenuity pathway analysis (IPA) software, we predicted that the differentially expressed peptides are associated with developmental disorder, inflammatory response, and organismal injury. And we preliminarily investigated the antiinflammatory effect of differentially expressed peptides on human lung epithelial cells. This study helps to broaden the knowledge of the hUC-MSC secretome at the peptide level and provided potential clues for the treatment of respiratory diseases in premature infants.

## Materials and methods

### Patients and samples

Umbilical cords were obtained from six infants without genetic or structural anomalies delivered at 27–41 weeks of gestation with parental written consent. Cases involving maternal diabetes, pre-eclampsia, eclampsia, intrauterine growth retardation (IUGR), or infectious diseases were excluded, because these factors may influence cell proliferation, cytokine expression, and other functions [29–33]. This study was approved by the Ethics Committee of Changzhou Maternal and Child Health Care Hospital (approval number 2019126) and conducted in accordance with the approved guidelines.

### Preparation of hUC-MSCs and CM

Human umbilical cords were collected after preterm (*n* = 3) or full term (n = 3) deliveries hUC-MSCs were obtained by the tissue explants adherent method, as previously reported [17, 34]. Each umbilical cord (about 10 cm) was washed in phosphate-buffered saline (PBS; Gibco, Grand Island, CA, USA) and with 1% penicillin/streptomycin (P/S; Gibco) to remove residual blood from the vein and arteries. After the cord was cut longitudinally, and the arteries and vein were removed, Wharton’s jelly was finely dissected into small pieces. The pieces were individually placed on 100-mm^2^ tissue culture dishes with Dulbecco’s modified Eagle medium/nutrient mixture F-12 (DMEM/F-12; Gibco) containing 10% fetal bovine serum (FBS; Gibco), and 1% P/S (Gibco) and incubated for 10–12 days at 37 °C with 5% CO_2_. The medium was subsequently exchanged every 2–3 days. The cultures were passaged when they reached 80–90% confluency after carefully removing the umbilical cord tissues. hUC-MSCs at passage 3 were cultured to 80–90% confluence in a T75 culture flask (about 10^6^ cells). The complete medium was replaced with serum-free DMEM/F-12 medium (5 ml) to avoid peptides contamination from FBS. The collected serum-free medium was centrifuged for 10 min at 300×*g* at 4 °C to remove cell debris, and protease inhibitor (Roche, Basel, Switzerland) was added. Lastly, the mixture was snap-frozen in liquid nitrogen and stored at − 80 °C until used.

### Peptide extraction and purification

Before peptide extraction, the protein integrity of the CM samples was appraised. The collected CM samples were concentrated by centrifugation under vacuum (LaboGene, Allerød, Denmark), boiled in sodium dodecyl sulfate (SDS)-sample buffer at 95 °C for 10 min and then subjected to 12% SDS-polyacrylamide gel electrophoresis (PAGE). The SDS-PAGE gel was stained using a Pierce Silver Stain Kit (Thermo Fisher Scientific, Waltham, MA, USA) following the manufacturer’s protocol. Thereafter, the samples were filtered through an ultrafiltration tube (Amicon Ultra-15, Millipore, MA, USA) with a molecular weight cutoff (MWCO) of 10 kDa to acquire the filtered liquid containing the peptides. The protein concentration of the supernatant was also measured using the Bradford protein assay [35].

### TMT labeling and LC-MS/MS analysis

The peptides from preterm and term hUC-MSC CM were reduced with 10 mM DL-dithiothreitol (DTT; Promega, WI, USA) for 1 h at 56 °C and alkylated with 55 mM iodoacetamide (Promega) for 1 h in the dark at RT. Thereafter, precooled acetone was added, and the peptides were precipitated over 3 h at − 20 °C. After centrifuging for 20 min at 20,000×*g* and 4 °C, the precipitate was dissolved in 300 μl of the following buffer: 50% triethylamine borane (Sigma) and 0.1% SDS (Sigma). Next, the peptide solution was desalted using a Strata-X C18 column (Phenomenex, Torrance, CA, USA) and dried and labeled with TMT reagent (TMT 6-plex Label Reagent; Thermo Fisher Scientific) for 1 h [36]. Next, the preterm and term samples were mixed at a 1:1 ratio on the basis of the total peptide amount. Analysis of labeled peptides was performed on a Q Exactive Orbitrap LC-MS/MS system (Thermo Fisher Scientific). Qualitative and relative quantitative analyses of the detected peptides were performed using the SWISSPROT_human database and Mascot software (version 2.3.01). Peptides with absolute fold change ≥ 1.5 and *P* value < 0.05 were considered differentially expressed.

### Bioinformatics analysis

The molecular weight (MW) and isoelectric point (PI) of the identified peptides were calculated using the online tool PI/MW (http://web.expasy.org/compute.pi/). A Gene Ontology (GO) analysis (http://www.blast2go.com/b2ghome) was carried out to explore the possible cellular components, biological processes, and molecular functions related to the precursor proteins. Diseases and regulator effects networks analysis of the differentially expressed peptides and their precursors were performed using ingenuity pathway analysis (IPA) software v7.1 (Ingenuity Systems, Mountain View, CA, USA) [37]. The UniProt database (http://www.uniprot.org/) was used to detect the predominant subcellular locations of the precursors of the differentially expressed peptides. The Open Targets Platform database (http://www.targetvalidation.org/) was applied to study the diseases associated with the protein precursors [38].

### Synthetic peptides

All the peptides used in this study were synthesized by GenScript (Nanjing, Jiangsu, China) through the solid-phase method. The purity of each peptide was 95% detected by HPLC-MS method. All the used peptides were preserved in freeze-drying at − 20 °C until immediately dissolved in aseptic water for cell treatment in vitro.

### Cell culture

Two peptides TGAKIKLVGT and AAAAGPANVH were selected with high fold change, and we investigated the effects on human lung epithelial cells A549 (Meiyan, Shanghai, China) stimulated by hydrogen peroxide (H_2_O_2_; Kelong, Chengdu, Sichuan, China). A549 cell is a human lung carcinoma cell line from an adult with similar characteristics of human alveolar basal epithelial cells, and was often used for researches in BPD. A549 cells were cultured in Dulbecco’s modified Eagle medium (Gibco) with 10% FBS and 1% P/S. The A549 cells were exposed to 1 mM H_2_O_2_ with or without peptides (1 μM, 10 μM, and 100 μM) for 24 h in serum-free DMEM with 1% P/S. The sample size was 3 biological independent samples per group.

### Quantitative real-time polymerase chain reaction (QRT-PCR)

Total RNA was extracted with Trizol reagent (Invitrogen; Thermo Fisher Scientific Inc., Shanghai, China) according to the manufacturer’s instructions. The RNA concentration was detected by ultraviolet spectrophotometer and the appropriate OD value at 260 to 280 nm was 1.8 to 2.0. Then, qRT-PCR was carried out to measure the gene expression levels of inflammatory factors: tumor necrosis factor-α (TNF-α), interleukin-1β (IL-1β), and interleukin-6 (IL-6) mRNAs in A549 cells using SYBR Green qPCR method (Thermo Fisher Scientific, Waltham, MA). Primer sequences were the following: human TNF-α: forward, 5′ CCTCTCTCTAATCAGCCCTCTG 3′, reverse, 5′ GAGGACCTGGGAGTAGATGAG 3′; human IL-1β: forward, 5′ AGCTACGAATCTCCGACCAC 3′, reverse, 5′ CGTTATCCCATGTGTCGAAGAA 3′; human IL-6: forward, 5′ ACTCACCTCTTCAGAACGAATTG 3′, reverse, 5′ CCATCTTTGGAAGGTTCAGGTTG 3′; and human β-actin: forward, 5′ AGCGAGCATCCCCCAAAGTT 3′, reverse, 5′ GGGCACGAAGGCTCATCATT 3′. To calculate fold change in the expression of these genes, ΔCt = Ct of individual genes − Ct of β-actin was first obtained. ΔΔCt = ΔCt of treated groups − ΔCt of control groups was then obtained. Fold change was calculated as 2−ΔΔCt, with control groups as 1.0-fold.

### Western blot analysis

Lysates from cultured cells were similarly prepared using a cell scraper. Homogenates were clarified by centrifugation (10,000×*g*, 4 °C, 10 min). Protein concentration was quantified with a BCA protein assay (Beyotime, Shanghai, China). Cell extracts resolved on a 10% or 12% reducing SDS-PAGE gel were transferred to a nitrocellulose membrane according to molecular weight. Blots were probed with the following antibodies: rabbit anti-TNF-α (1:2000; Proteintech, Wuhan, China), anti-IL-1β (1:1000; Proteintech, Wuhan, China), and mouse anti-IL-6 (1:5000; Proteintech, Wuhan, China), whereas mouse GAPDH (1:5000; Proteintech, Wuhan, China) served as a loading control.

### Enzyme-linked immunosorbent assay (ELISA)

ELISA was also performed to detect the expression levels of inflammatory factors in A549 cells by ELISA kits for human TNF-α, IL-1β, and IL-6 (4A Biotech, Beijing, China), according to the manufacturer’s instructions. Optical density was measured at 450 nm using an ELISA microplate reader. No significant cross-reactivity or interference was observed.

### Statistical analysis

Student’s *t* test or one-way ANOVA was employed for statistical comparisons. The results of the bioinformatics analysis were visualized using GraphPad Prism 5/7 software. The statistical significances were calculated as *P* values, and *P* < 0.05 was considered statistically significant.

## Results

### Isolation and characterization of MSCs derived from human umbilical cords

The range of gestational ages (GA) was 31–32 weeks of gestation for the preterm umbilical cords and 40–41 weeks for the term cords (Table 1). The hUC-MSCs (obtained by the tissue explants adherent method) had a typical fibroblast phenotype (Fig. S1A). Both preterm and term hUC-MSCs were positive for CD29, CD73, and CD105 staining and negative for CD31, CD34, and HLA-DR staining (Fig. S1B). Furthermore, these isolated cells had the potential to differentiate into adipocytes and chondrocytes (Fig. S1A). These results confirmed that MSCs from human umbilical cords were successfully isolated, without significant differences in morphology, expression of cell surface markers or differentiation capacities between the preterm and term groups.

**Table 1:** Cohort clinical date of hUC-MSC samples

Sample	Gestational age (weeks)	Birth weight (g)	Sex	Apgar score 1 min	Apgar score 5 min	Maternal age (years)	Gravidity	Parity	Perinatal history
P 1	32 + 5	1630	Female	10	10	28	2	1	Cesarean section due to placental abruption
P 2	32 + 5	1850	Male	10	10	29	2	1	Vaginal delivery due to premature rupture of fetal membranes
P 3	31	1600	Female	8	8	33	5	3	Cesarean section due to active premature labor
T 1	40 + 3	4670	Male	10	10	23	1	1	Cesarean section due to primary uterine atony
T 2	41 + 1	3360	Female	10	10	29	2	2	Repeated cesarean section
T 3	40 + 6	4250	Female	10	10	27	2	1	Cesarean section due to non-reassuring fetal status

### Identification of differentially expressed peptides in hUC-MSC CM from preterm and term infants

**Fig. 1:**
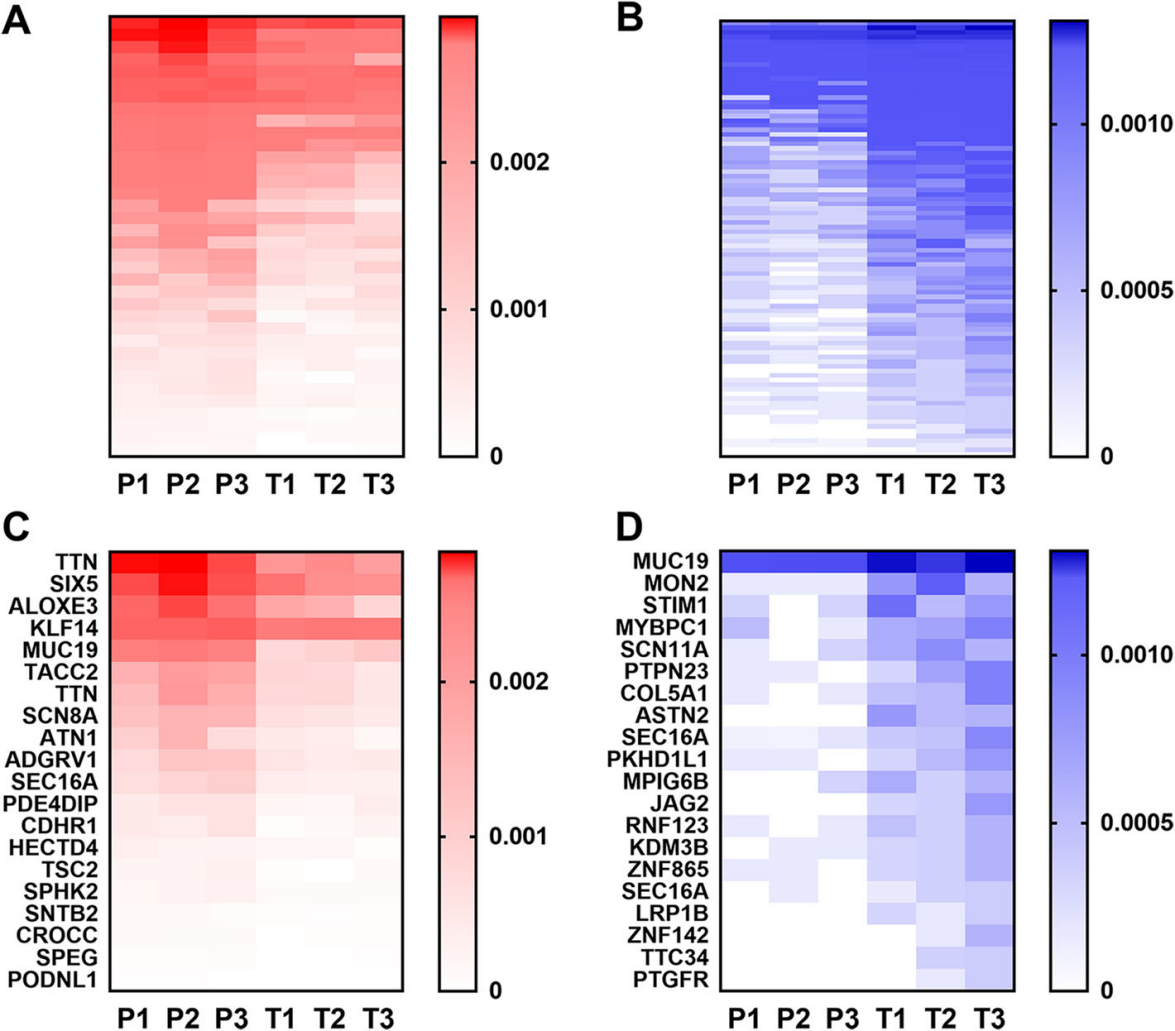
Differentially expressed peptides in hUC-MSC conditioned medium (CM) from preterm infants compared with term infants. **a**, **b** Upregulated and downregulated peptides visualized using heatmaps (*n* = 3 per group, P1–3 represent preterm infants and T1–3 represent term infants). **c**, **d** Top 20 upregulated and top 20 downregulated peptides visualized using heatmaps

We verified the protein integrity of the hUC-MSC CM by silver staining (Fig. S2). The peptides from preterm and term hUC-MSC CM were directly analyzed by the TMT labeling method combined with LC-MS/MS. We identified a total of 3099 peptides in hUC-MSC CM from both groups. A total of 131 peptides were observed to be significantly differentially expressed (absolute fold change ≥ 1.5, *P* value < 0.05) in the hUC-MSC CM from the preterm group compared with the term group, comprising 37 upregulated peptides (Fig. 1a) and 94 downregulated peptides (Fig. 1b). The top 20 upregulated and top 20 downregulated peptides are shown with their precursor proteins in Fig. 1c and d. All the differentially expressed peptides are shown in Table 2.

**Table 2:** Differentially expressed peptides in hUC-MSC conditioned medium from preterm and term infants

Accession	Gene	Protein	Peptide	MW (kD)	Fold change	− 10lgP
Upregulated peptides						
Q6PEZ8-3	PODNL1	Podocan-like protein 1	PSLERLHLQNNLISKVPR	5.94	∞	6.96
A0A0A0MTS7	TTN	Titin	ESDSG	2.92	13.62	9.66
Q9BYJ1-2	ALOXE3	Hydroperoxide isomerase ALOXE3	LNGRQQY	0.54	7.95	10.08
Q15772-1	SPEG	Striated muscle preferentially expressed protein kinase	SCTVAVARVPGKLAPPEVPQ	0.76	5.96	10.05
Q8N196	SIX5	Homeobox protein SIX5	AAAAGPANVH	1.00	5.93	11.82
P49815-3	TSC2	Tuberin	PAGPAVRL	0.57	4.52	10.67
A0A0A6YYA3	CDHR1	Cadherin-related family member 1	RVLRKRPSPAPRTIRIE	0.69	3.82	9.34
Q7Z5P9-2	MUC19	Mucin-19	DDFMSSQN	2.05	3.46	5.01
P54259	ATN1	Atrophin-1	GPARPYHP	0.67	3.28	11.27
A0A140TA73	SNTB2	Beta-2-syntrophin	NGLPNGGGAGDS	0.88	2.98	7.03
Q9UQD0-2	SCN8A	Sodium channel protein type 8 subunit alpha	EAGID	1.81	2.67	16.18
Q5TZA2-2	CROCC	Rootletin	RLLKGEASLEV	0.68	2.67	9.78
E7EMZ9	TACC2	Transforming acidic coiled-coil-containing protein 2	RMSESPTPC	1.29	2.57	8.92
A0A075B756	KLF14	Krueppel-like factor 14	TKHARRHP	0.94	2.52	17.50
A0A0A0MTS7	TTN	Titin	LEDGG	3.06	2.50	12.31
O15027-2	SEC16A	Protein transport protein Sec16A	KSILTQ	2.31	2.49	13.78
Q9NRA0-3	SPHK2	Sphingosine kinase 2	EWDGIVTVSGDGLLHEVLN	0.56	2.46	10.14
Q8WXG9	ADGRV1	Adhesion G-protein coupled receptor V1	EAGLD	2.02	2.23	19.57
Q9Y4D8	HECTD4	Probable E3 ubiquitin-protein ligase HECTD4	KLAKLQRIARQAVAALCALGG	1.02	2.21	6.58
A0A087WVF8	PDE4DIP	Myomegalin	QSMMAV	1.01	2.18	21.26
Q01167	FOXK2	Forkhead box protein K2	QTVHVVH	0.67	2.18	12.99
Q5IJ48	CRB2	Protein crumbs homolog 2	LLEVAVPAACACLLLLLLGLLSGILAARK	0.78	2.18	5.08
Q9ULE3	DENND2A	DENN domain-containing protein 2A	FLHKK	1.70	2.17	21.46
P53420	COL4A4	Collagen alpha-4(IV) chain	PGEPGLVGPPGQPGRPG	0.84	2.14	7.51
Q7Z5P9-2	MUC19	Mucin-19	FLGGS	2.11	2.11	13.79
P01833	PIGR	Polymeric immunoglobulin receptor	QADGSRASVD	0.49	2.10	5.11
Q09666	AHNAK	Neuroblast differentiation-associated protein AHNAK	KLKGDI	1.57	2.08	15.35
P36776	LONP1	Lon protease homolog, mitochondrial	KHKPR	0.99	2.07	8.69
A0A0A0MTS7	TTN	Titin	VPEAPKEVVPEKKVPVTPPKK	2.94	1.98	10.42
Q8NEZ4	KMT2C	Histone-lysine N-methyltransferase 2C	QQNNLSNP	1.03	1.96	10.44
A0A0A0MTS7	TTN	Titin	SPPSP	2.94	1.93	11.70
Q7Z5P9-2	MUC19	Mucin-19	AGTSI	2.24	1.89	23.15
O75592-2	MYCBP2	E3 ubiquitin-protein ligase MYCBP2	QLLYR	2.05	1.79	11.10
Q9H6K5-2	PRR36	Proline-rich protein 36	PPSLQTLPSPPATPPSQVPPTQ	0.81	1.78	9.94
P08913	ADRA2A	Alpha-2A adrenergic receptor	ISAVISFPPLISIEKKGGGG	0.93	1.71	9.59
Q9UJ55	MAGEL2	MAGE-like protein 2	PPPIRPGP	1.10	1.65	15.90
Q4V328-4	GRIPAP1	GRIP1-associated protein 1	LCSQMEQLE	0.63	1.60	6.91
Downregulated peptides						
O14526-3	FCHO1	F-BAR domain only protein 1	AGIVRVF	0.53	−1.56	9.90
Q7Z5P9-2	MUC19	Mucin-19	KTLAAGS	2.15	−1.57	14.06
Q99814	EPAS1	Endothelial PAS domain-containing protein 1	TPLSSMGGRS	1.00	−1.58	18.34
Q6ZNL6	FGD5	FYVE, RhoGEF and PH domain-containing protein 5	EDHAQ	0.77	−1.58	13.24
Q9BW04-2	SARG	Specifically androgen-regulated gene protein	LTTPKPRKLPPN	0.61	−1.60	5.40
O15027-2	SEC16A	Protein transport protein Sec16A	QACAASGS	2.41	−1.61	17.59
S4R393	ZSWIM8	Zinc finger SWIM domain-containing protein 8	QTHKPQT	0.99	−1.64	13.03
Q2VWA4	SKOR2	SKI family transcriptional corepressor 2	GGSGGDCSAG	0.50	−1.65	8.13
Q9Y6V0-6	PCLO	Protein piccolo	QQPGPAKPPP	1.00	−1.69	6.21
P28329	CHAT	Choline O-acetyltransferase	GLPKLPVPPLQQ	0.66	−1.70	5.12
Q8WXH0	SYNE2	Nesprin-2	KIYKKFLKKAQDLTSLLKEL	2.04	−1.71	5.55
P13611-5	VCAN	Versican core protein	QPEFSS	1.97	−1.74	29.72
Q8TE85	GRHL3	Grainyhead-like protein 3 homolog	LFIPNVHFSSLQRSG	0.54	−1.78	9.85
A0A1B0GUF7	IQCM	IQ domain-containing protein M	KTFKT	0.88	−1.79	14.67
A0A087WXW9	COL5A1	Collagen alpha-1(V) chain	PPGEV	2.70	−1.83	8.75
A0A0A0MTS7	TTN	Titin	KACDPVF	2.92	−1.87	8.46
A0A0A0MTS7	TTN	Titin	IVASDVTKRLIKANLLANN	2.78	−1.87	5.62
H0Y5I7	SFI1	Protein SFI1 homolog	QQLAARRQEQRATVRALW	0.82	−1.88	6.99
A6NMZ7	COL6A6	Collagen alpha-6(VI) chain	RRAIN	0.91	−1.89	13.43
Q8NEZ4	KMT2C	Histone-lysine N-methyltransferase 2C	EGCVK	1.08	−1.89	12.10
A0A087WXW9	COL5A1	Collagen alpha-1(V) chain	GPRGITGKPGPK	2.70	−1.90	10.79
Q9Y6W6	DUSP10	Dual specificity protein phosphatase 10	DNQAQT	1.21	−1.91	9.85
A0A0J9YXV3	N/A	Uncharacterized protein	KIGLGY	0.94	−1.91	14.05
A0A0A0MTS7	TTN	Titin	EGNKDD	3.08	−1.91	12.34
Q92616	GCN1	eIF-2-alpha kinase activator GCN1	ILDVASLEVLN	0.66	−1.92	5.74
Q9NR09	BIRC6	Baculoviral IAP repeat-containing protein 6	DNESCTN	1.47	−1.95	6.90
Q8TEP8	CEP192	Centrosomal protein of 192 kDa	LLSTTK	1.70	−1.96	17.83
A0A0A0MTS7	TTN	Titin	DPPGKPVPLN	3.22	−2.08	14.35
Q8TE73	DNAH5	Dynein heavy chain 5, axonemal	QRVKSKIPAAIEQLIVPHLAKVDEALQPGLAAL	1.84	−2.10	7.15
P10827-4	THRA	Thyroid hormone receptor alpha	LHARAV	0.51	−2.11	9.87
Q9P2D3	HEATR5B	HEAT repeat-containing protein 5B	HAKGK	0.83	−2.12	12.50
O60423-3	ATP8B3	Phospholipid-transporting ATPase IK	YGLVI	0.98	− 2.13	11.77
P06401-2	PGR	Progesterone receptor	GPLLKGKPRALGGAAAGGG	0.77	− 2.14	5.47
A0A140T8Y3	TNXB	Tenascin-X	HGRGRCEEGRCLCDPGYTGPTCATRMCPADCRGRGRCVQGVCLCHVGYGGEDCGQ	1.71	−2.14	5.92
P27658	COL8A1	Collagen alpha-1(VIII) chain	GIDGVKPPHAYGAKKGKN	0.65	−2.14	6.82
Q9C093	SPEF2	Sperm flagellar protein 2	ESLCEKVKEILTTEIAKKKN	0.69	−2.14	7.26
Q8NAC3-3	IL17RC	Interleukin-17 receptor C	AAALSLILLLKKDHAKGWLRLLKQ	0.48	−2.15	5.00
Q92771	DDX12P	Putative ATP-dependent RNA helicase DDX12	KGGLLGRLAARKKIFQEPK	0.67	−2.16	6.09
Q6PJG9	LRFN4	Leucine-rich repeat and fibronectin type-III domain-containing protein 4	VAVGGVLVAALLVFTVALLVRGRGAGNGRL	0.69	−2.17	5.32
Q9HD67	MYO10	Unconventional myosin-X	KTSCVE	0.82	−2.23	14.68
Q96DN2	VWCE	von Willebrand factor C and EGF domain-containing protein	RPVLHLLQLLLRTNLMKTQTL	0.50	−2.26	9.34
Q96QD8	SLC38A2	Sodium-coupled neutral amino acid transporter 2	VFNLSNAIVGSGILGLS	0.60	−2.31	12.68
A0A0C4DGG6	NPC1L1	NPC1-like intracellular cholesterol transporter 1	VFAVVTILLVGFRVAPARDKSKMVDPKK	0.68	−2.32	12.53
Q9UQD0-2	SCN8A	Sodium channel protein type 8 subunit alpha	VSLVSLIAN	1.71	−2.33	12.11
Q9UPA5	BSN	Protein bassoon	KGGPRPR	2.05	−2.34	10.76
Q9UKV8	AGO2	Protein argonaute-2	KLQAN	1.17	−2.36	13.86
Q9UQD0-2	SCN8A	Sodium channel protein type 8 subunit alpha	YLALL	1.72	−2.36	14.90
I6L894	ANK2	Ankyrin-2	KHKLNVP	0.89	−2.38	11.67
O95996	APC2	Adenomatous polyposis coli protein 2	PAAEAATKKPLPPLRH	0.78	−2.39	5.35
P36776	LONP1	Lon protease homolog, mitochondrial	TIAAKRAGVT	0.99	−2.46	12.42
H7BXZ5	KALRN	Kalirin	VKKCIHKATRKDVAVKFVSKKMKKKEQA	0.91	−2.46	9.58
Q8NF91-4	SYNE1	Nesprin-1	QKAVDHRKAIILSIN	1.02	−2.56	8.45
J3KQC6	TMPRSS13	Transmembrane protease serine 13	LPLIGCVLLLIALVVSLIILFQFW	0.45	−2.60	5.00
Q8TE73	DNAH5	Dynein heavy chain 5, axonemal	AQTKRLVGDVLLATAFLSYSGP	1.82	−2.62	5.48
I3L2R4	SLC2A4	Solute carrier family 2 (Facilitated glucose transporter), member 4, isoform CRA_b	IGAGVVNTVFTLVSVLLVERAGRRTLHLLGLA	0.62	−2.63	7.43
P42167	TMPO	Lamina-associated polypeptide 2, isoforms beta/gamma	KSEKTKKGRSIPVWIKILLFVVVAV	0.49	−2.66	6.27
Q9BW11-4	MXD3	Max dimerization protein 3	GPIHRRK	0.50	−2.66	7.43
E7EPG1	MMRN1	Multimerin-1	LPDIQLLQKGLTEFV	0.66	−2.70	6.46
G5EA42	TMOD2	Tropomodulin 2 (Neuronal), isoform CRA_a	HVKKF	0.67	−2.76	12.93
A0A0A0MTS7	TTN	Titin	SSRLECKI	2.84	−2.77	7.13
Q7Z5P9-2	MUC19	Mucin-19	QNGIIVI	2.18	−2.78	18.23
Q5H8A4	PIGG	GPI ethanolamine phosphate transferase 2	WLAAGGVMVLASALLCVIVSVLTNVLVGGN	1.18	−2.80	9.72
Q9NR09	BIRC6	Baculoviral IAP repeat-containing protein 6	TRIGLKLIDILLRNCAAS	1.36	−2.82	5.14
A0A0A0MTS7	TTN	Titin	VVHAGGVIRIIAYV	2.83	−2.89	5.92
Q8NG04	SLC26A10	Solute carrier family 26 member 10	EPVVKALTSGAALHVLLSQLPSLLGLSL	0.59	−2.91	11.40
J3KNF3	TET3	Methylcytosine dioxygenase TET3	GPEGCSA	1.65	−2.94	15.44
Q92835-2	INPP5D	Phosphatidylinositol 3,4,5-trisphosphate 5-phosphatase 1	PLPVKSPA	1.01	−2.97	22.11
Q9Y6R1	SLC4A4	Electrogenic sodium bicarbonate cotransporter 1	HHTIYIGVHVPKSYR	0.78	−2.99	8.24
Q99707	MTR	Methionine synthase	KSARVMKKAVG	1.66	−3.12	21.33
Q8WXG9	ADGRV1	Adhesion G-protein coupled receptor V1	RFLQSIYLVPEEDHILIIPVVRGKDN	1.97	−3.13	8.73
P43243-2	MATR3	Matrin-3	HLILN	0.67	−3.21	8.71
O75970-3	MPDZ	Multiple PDZ domain protein	FISLLKT	0.58	−3.22	11.08
Q96N23-2	CFAP54	Cilia- and flagella-associated protein 54	HLKKPKIKISGSPLTLKPPLRRSSSVKET	0.62	−3.25	6.96
F8WBW8	BAHCC1	BAH and coiled-coil domain-containing protein 1	RSVKAKVGTTL	1.16	−3.27	15.77
F8VZY0	MYBPC1	Myosin-binding protein C, slow-type	TDAKIFVRVKAVNAAGAS	0.76	−3.39	9.25
G0XQ39	STIM1	STIM1L	GVHPGSLVEKLPDSPALAKKALLALNHGL	0.59	−3.56	12.45
P0CJ78	ZNF865	Zinc finger protein 865	MEANPAGSGAGGGGSSGIGGEDGVHFQSYPFDFLEFLNHQRFEPMELYGEHAKAVAA	0.86	−3.84	11.26
Q7LBC6-2	KDM3B	Lysine-specific demethylase 3B	VKSKASLPN	0.69	−3.87	7.94
Q9UI33-3	SCN11A	Sodium channel protein type 11 subunit alpha	IGAIPAILNV	0.83	−4.13	15.16
Q5XPI4	RNF123	E3 ubiquitin-protein ligase RNF123	HYLRLTIAI	0.67	−4.16	8.06
O15027-2	SEC16A	Protein transport protein Sec16A	AGSLCQALLPGPSNEAAGDVWGDTASTGVPDASGSQYE	2.30	−4.48	5.51
B7ZLJ5	MPIG6B	C6orf25 protein	YPQLLIPLLGAGLVLGLGALG	0.48	−4.66	5.03
Q86WI1	PKHD1L1	Fibrocystin-L	LFVGR	1.16	−4.97	17.10
Q7Z3U7	MON2	Protein MON2 homolog	KPPQYGQLETKHIAN	1.01	−5.28	21.73
F1T0I1	SEC16A	Protein transport protein sec16	RRRAN	2.30	−5.79	11.96
A0A087WXW9	COL5A1	Collagen alpha-1(V) chain	HPGLI	2.50	−5.82	5.85
Q9H3S7	PTPN23	Tyrosine-protein phosphatase non-receptor type 23	KLELLRQN	0.65	−6.11	5.79
Q7Z5P9-2	MUC19	Mucin-19	TGAKIKLVGT	2.11	−6.37	6.06
P52746	ZNF142	Zinc finger protein 142	TGLKP	4.94	-∞	13.46
P43088-7	PTGFR	Prostaglandin F2-alpha receptor	QKSKASFLL	3.34	-∞	9.46
A0A1C7CYW7	TTC34	Tetratricopeptide repeat protein 34	TGGQRLLAAL	3.68	-∞	12.39
Q9Y219	JAG2	Protein jagged-2	CGSDAGPGMPGTAASGVCGPHGRCVSQPGGN	3.52	-∞	10.83
E7ERG8	LRP1B	Low-density lipoprotein receptor-related protein 1B	QVDQFSCGNGRCIPRAWLCDREDDCGDQTDEMASCEFPTCEPLT	3.26	-∞	5.58
O75129-2	ASTN2	Astrotactin-2	TCHLC	5.80	-∞	12.93

### Basic characteristics of the differentially expressed peptides in hUC-MSC CM from preterm and term infants

**Fig. 2:**
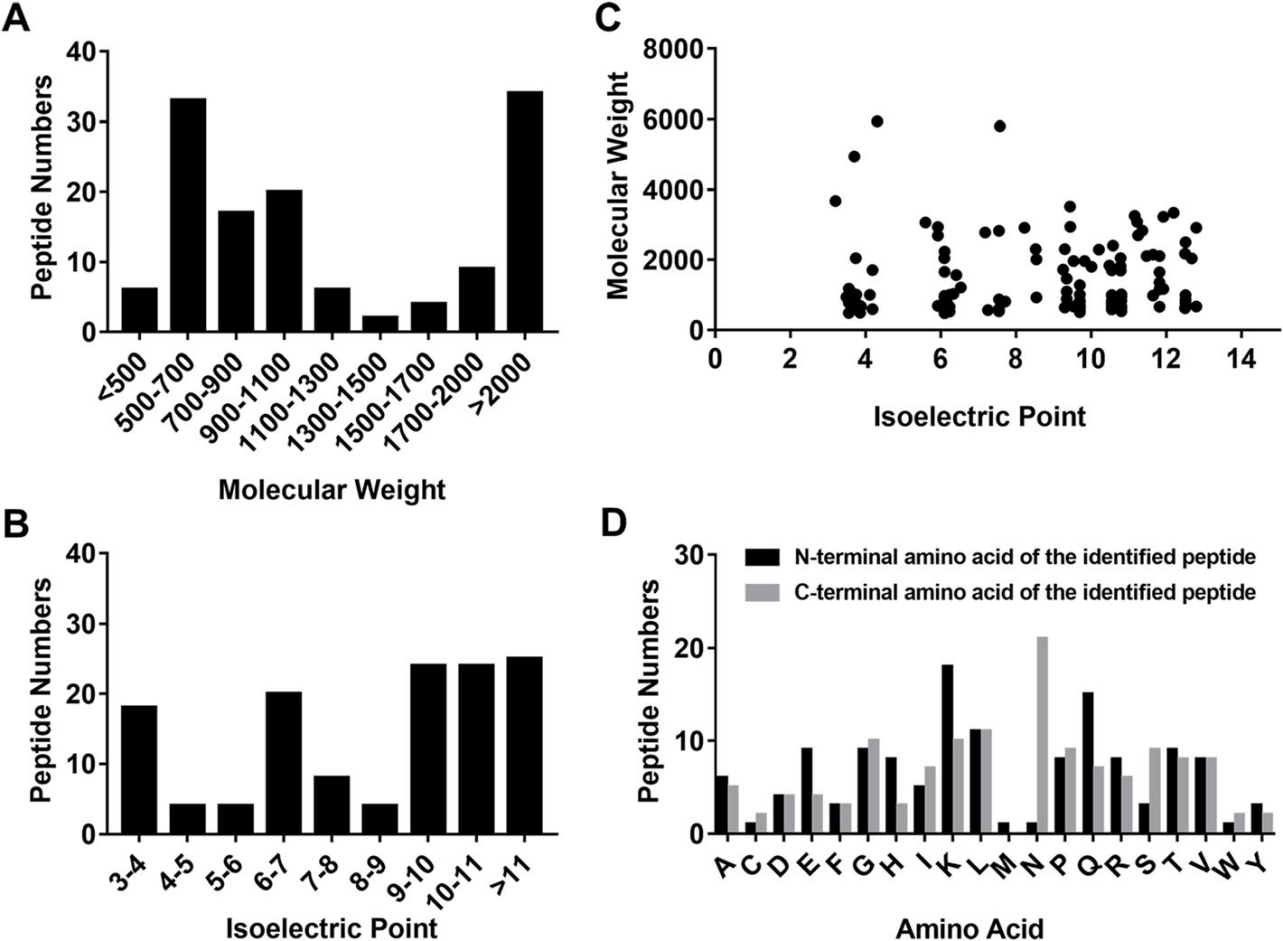
Basic features of the differentially expressed peptides in hUC-MSC CM from preterm and term infants. **a**, **b** Molecular weights (MW) and isoelectric points (PI) of the differentially expressed peptides. **c** Scatter plot of MW versus PI of the differentially expressed peptides. **d** Distributions of the N- and C-terminals of the differentially expressed peptides

The MW and PI of the differentially expressed peptides were analyzed. The MW of most peptides ranged from 500 to 700 Da (Fig. 2a), and the PI ranged from 3 to 11 (Fig. 2b). We also investigated the distribution of the MW relative to the PI (Fig. 2c). Peptides are cleaved from their precursor proteins by specific enzymes [37], so we analyzed the cleavage sites at the N- and C-terminals of the identified peptides. Lysine (K) was the most common N-terminal amino acid (accounting for 13.7% of the peptides), while asparagine (N) was the most common C-terminal amino acid (accounting for 16.0% of the peptides) (Fig. 2d).

### GO analysis and subcellular location analysis of the differentially expressed peptide precursors

**Fig. 3:**
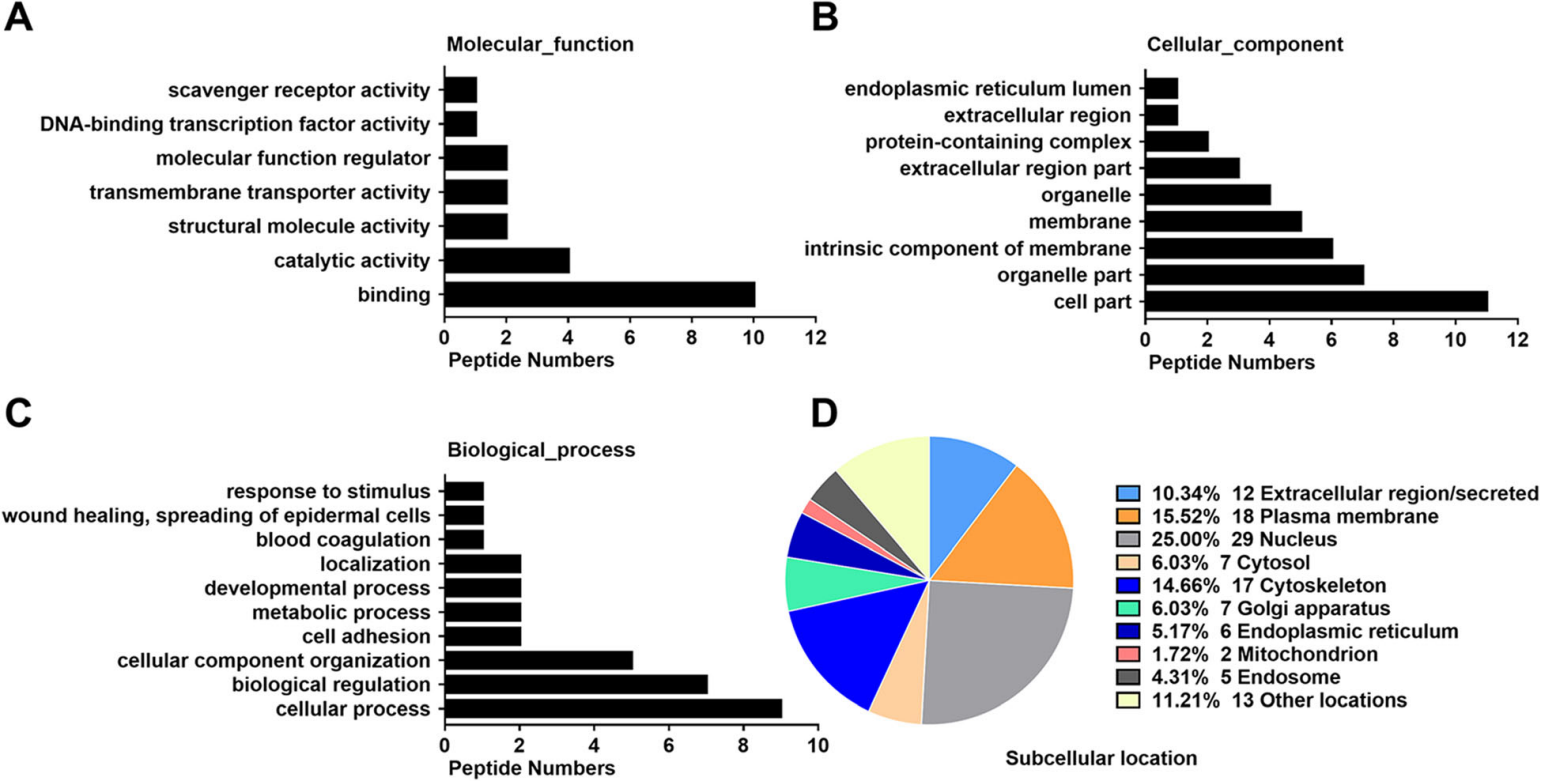
Gene Ontology (GO) and subcellular location analysis of the differentially expressed peptide precursors. **a** Molecular functions. **b** Cellular components. **c** Biological processes. **d** Subcellular locations

Next, molecular functions, cellular components and biological processes of the corresponding precursor proteins were determined by GO analysis to predict the latent functions of the differentially expressed peptides. Binding and catalytic activity were the most highly enriched molecular functions (Fig. 3a). Cell part, organelle part, and intrinsic component of membrane were the most highly enriched cellular components (Fig. 3b). Cellular process, biological regulation, and cellular component organization were the most highly enriched biological processes (Fig. 3c). Furthermore, we categorized the subcellular locations of the precursor proteins of all 131 peptides in accordance with their annotations in the UniProt database. The analysis revealed that the nucleus (25%), plasma membrane (16%), and cytoskeleton (15%) were the predominant subcellular locations of the differentially expressed peptide precursors. About 10% of the precursors were types of proteins that are located in the extracellular region of hUC-MSCs (Fig. 3d).

### Diseases and regulator effects networks associated with the differentially expressed peptide precursors

**Fig. 4:**
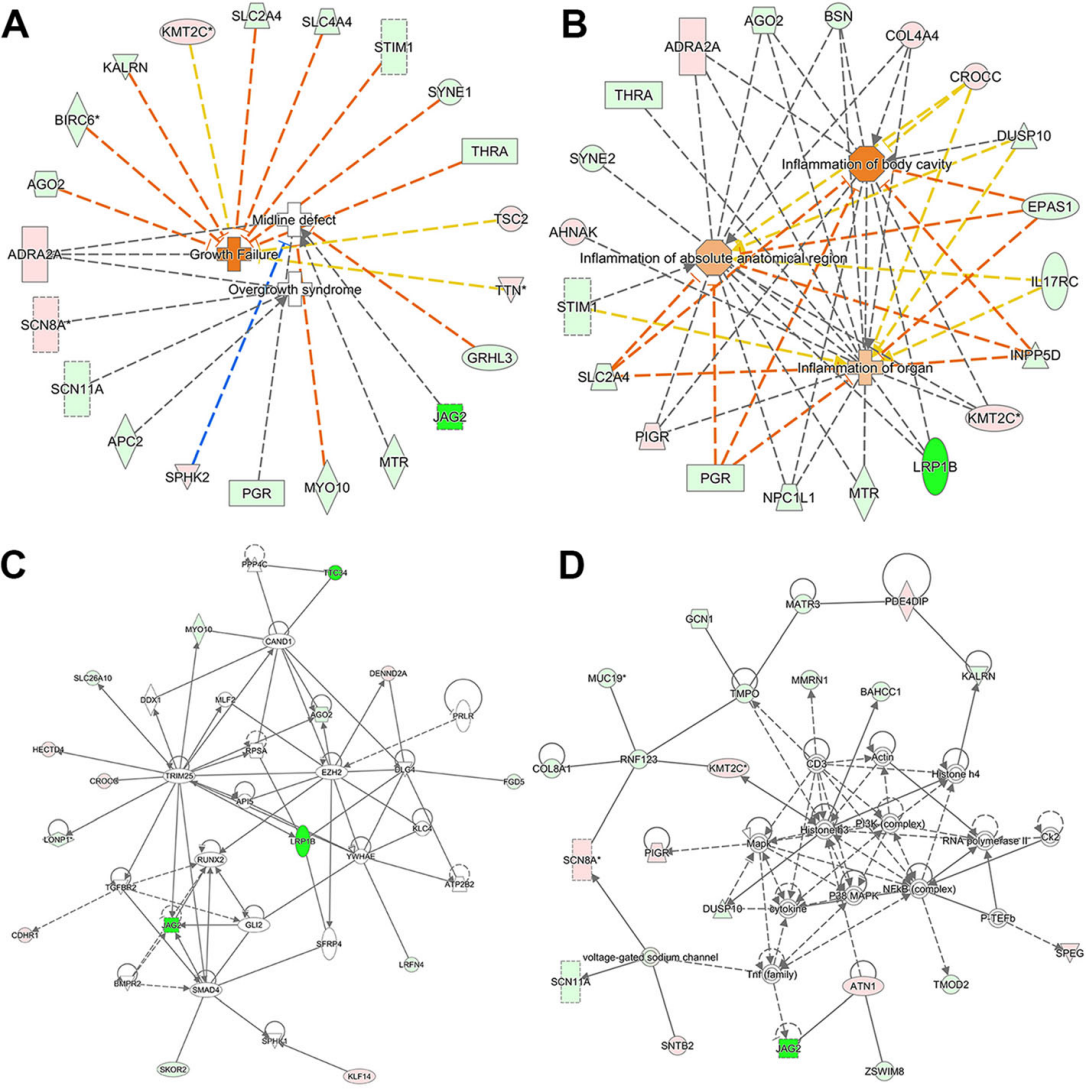
Diseases and regulator effects networks associated with the differentially expressed peptide precursors. Functional proteins related to **a** developmental disorder and **b** inflammatory response. Regulator effects network related to **c** cellular development, embryonic development, and organismal development and **d** organismal injury and abnormalities. Precursor proteins, diseases, and functions are shown as nodes, and the biological relationships between nodes are represented as lines with arrows. All lines are supported by at least one literature reference from the ingenuity pathway analysis (IPA) analysis. The intensity of the node color indicates the degree of upregulation (red) or downregulation (green)

We further evaluated the diseases and regulator effects networks associated with the differentially expressed peptide precursors using IPA software. Disease and functional protein network analysis indicated that several precursor proteins were involved in developmental disorders and inflammatory responses (Fig. 4a, b). More precisely, precursor proteins including Alpha-2A adrenergic receptor (ADRA2A), Protein argonaute-2 (AGO2), Baculoviral IAP repeat-containing protein 6 (BIRC6), Kalirin (KALRN), and Histone-lysine N-methyltransferase 2C (KMT2C) were involved in developmental disorders, and KMT2C, Solute carrier family 2 (SLC2A4), Electrogenic sodium bicarbonate cotransporter 1 (SLC4A4), STIM1L (STIM1), and Rootletin (CROCC) were involved in inflammatory responses. All the putative precursor proteins associated with diseases are shown in Table S1. Furthermore, the regulator effects network analysis showed that some of the protein precursors participated in the networks of cellular development, embryonic development, organismal development, and organismal injury and abnormalities (Fig. 4c, d). For example, AGO2, CROCC, DENN domain-containing protein 2A (DENND2A), Krueppel-like factor 14 (KLF14), and Lon protease homolog (LONP1) were involved in the networks of cellular development, embryonic development, and organismal development. Additionally, Atrophin-1 (ATN1), Collagen alpha-1 (VIII) chain (COL8A1), Protein jagged-2 (JAG2), KMT2C, and Mucin-19 (MUC19) were related to the network of organismal injury and abnormalities. All the precursor proteins involved in regulator effects networks are shown in Table S2.

### Putative bioactive peptides associated with respiratory diseases

It is well known that peptides with biological functions have functions that are related to the functions of their precursor proteins, with domains playing key roles in the biological functions [39, 40]. The UniProt database was used to analyze the peptides and their precursors and the results showed that 25 peptides were located in the functional domains of their corresponding precursors (Table S3). The preceding results suggested that these precursors are mainly associated with inflammatory responses and abnormal organ development, which contribute to premature infant respiratory diseases. Therefore, we focused on peptides and their precursors related to respiratory diseases. Using the Open Targets Platform database, we investigated whether these peptides might play potential roles in respiratory diseases. All told, 17 precursor proteins were found to be closely related to respiratory diseases (association score ≥ 0.5) (Table S4).

### TNF-α, IL-1β, and IL-6 in H_2_O_2_-treated lung epithelial cells with differentially expressed peptides

**Fig. 5:**
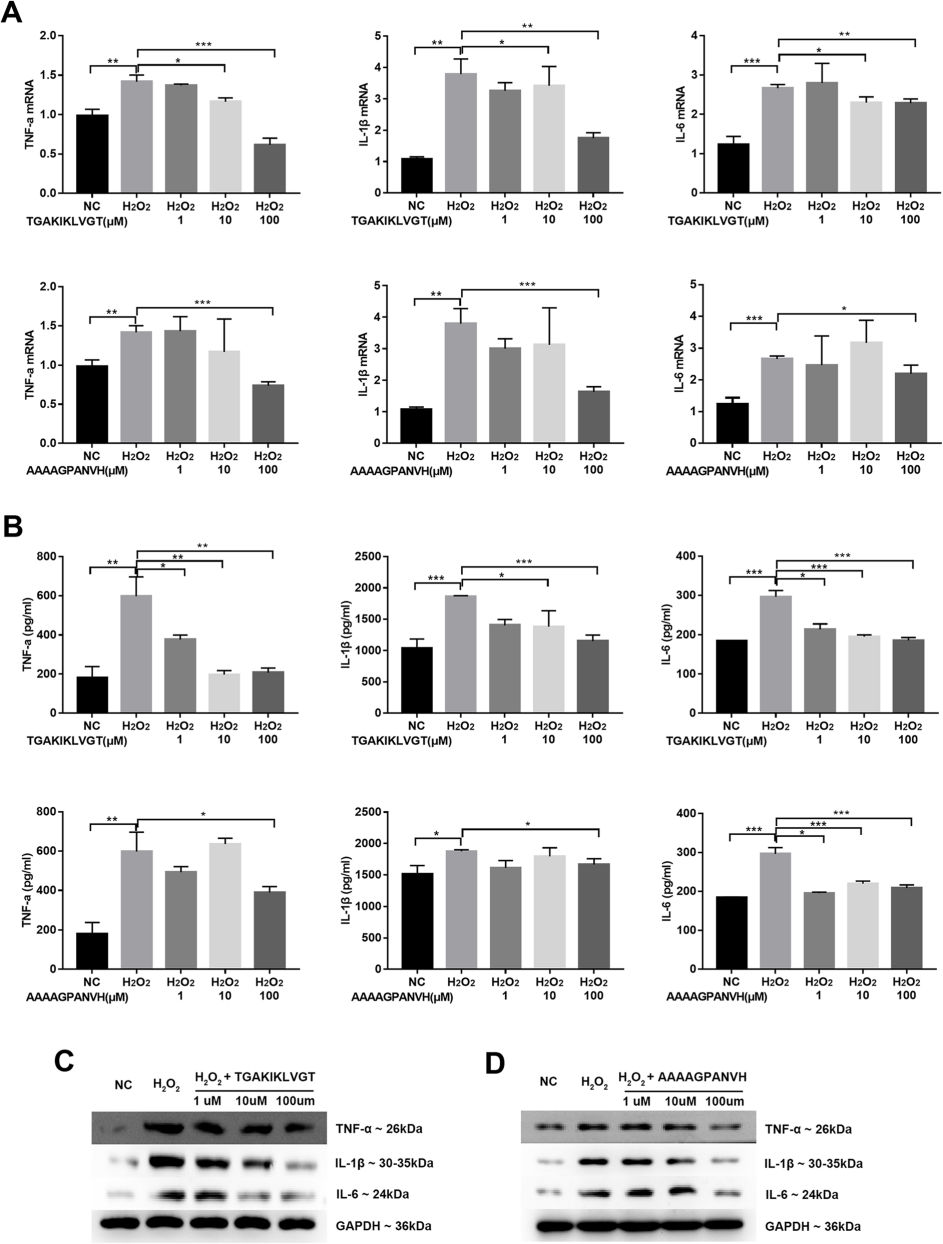
Function analysis of differentially expressed peptides in vitro. (**a**) TNF-α, IL-1β, and IL-6 mRNA expression assessed by qRT-PCR in A549 cells stimulated by H_2_O_2_ (1 mM) with or without peptides ^7118^TGAKIKLVGT^7127^ (MUC19) and ^508^AAAAGPANVH^517^ (SIX5) at concentrations of 1, 10, and 100 μM. **b** The protein levels of TNF-α, IL-1β, and IL-6 measured by ELISA analyze in H_2_O_2_-treated A549 cells with or without peptides. **c**, **d** The protein levels of TNF-α, IL-1β, and IL-6 measured by western blot in H_2_O_2_-treated A549 cells with or without peptides (*n* = 3 biological independent samples per group in qRT-PCR and WB, technical replication = 3 in ELISA. *P < 0.05, **P < 0.01, ***P < 0.001)

Previous researches have reported that lung epithelial cells A549 can be stimulated by H_2_O_2_ to induce inflammatory response [41, 42]. Therefore, we explored the effect of differentially expressed peptides on inflammatory response of H_2_O_2_-treated A549 cells. As observed, TNF-α, IL-1β, and IL-6 mRNA and protein expression levels were higher in A549 cells from the H_2_O_2_ (1 mM) group compared with the control group (Fig. 5), respectively, measured by qRT-PCR, ELISA, and western blot. According to the high fold change, we selected two peptides ^508^AAAAGPANVH^517^ derived from Homeobox protein SIX5 (SIX5, absolute fold change: 5.9) and ^7118^TGAKIKLVGT^7127^ derived from MUC19 (absolute fold change, 6.3) for future study. Peptide ^7118^TGAKIKLVGT^7127^ (MUC19) at 10 and 100 μM significantly attenuated the H_2_O_2_-induced increase of TNF-α, IL-1β, and IL-6. Moreover, we observed that the levels of inflammatory cytokines decreased more obviously with the ^7118^TGAKIKLVGT^7127^ at the concentration of 100 μM than 10 μM (Fig. 5). Meanwhile TNF-α, IL-1β, and IL-6 were significantly reduced in H_2_O_2_-treated A549 cells with peptide ^508^AAAAGPANVH^517^ derived from SIX5 at concentration of 100 μM, but not at 1 μM or 10 μM (Fig. 5). Both the two peptides help to suppress inflammatory response in H_2_O_2_-treated A549 cells.

## Discussion

The MSC secretome and its therapeutic effects have been extensively demonstrated in preterm diseases, such as BPD [43] and NEC [19]. Previously, most researchers considered soluble factors and extracellular vesicles as the primary components of the secretome derived from MSCs [44–46]. However, the paracrine substances from MSCs are not limited to these biomolecules. Therefore, further studies are required to explore more types of components derived from MSCs and investigate their potential functions.

With the advance of detection technologies, mounting evidence has confirmed the differences in hUC-MSCs between preterm and term groups, which may help to identify the possible regulators or mechanisms underlying MSC function. To compare the global gene expression patterns in hUC-MSCs between these two groups, Iwatani et al. used microarray analysis and revealed that upregulated WNT2B in preterm hUC-MSCs was involved in the control of hUC-MSC proliferation [47]. A very recent study comparing hUC-MSC transcriptomics and proteomics profiles from term and preterm groups showed that Frizzled-2 (FZD2) protein and mRNA expression levels were both higher in preterm hUC-MSCs [48]. Importantly, FZD2 is the receptor of Wnt5a/b and FZD2 mutations influence Wnt signaling, which mediates the epithelial to mesenchymal transition (EMT) during lung development [49]. In addition, by comparing the proteome of microvesicles collected from hUC-MSC CM between preterm and term groups, Bruschi et al. found that 173 proteins were significantly changed, 163 of which were increased in the preterm group [50]. However, there have been no comprehensive comparisons of hUC-MSC CM peptidomic profiles between preterm and term infants. In the present study, we found that 131 peptides derived from 106 precursor proteins were differentially expressed in the preterm hUC-MSC CM compared with the term group by TMT labeling quantification (Fig. 1). Our study provides hUC-MSC CM polypeptide profiles for preterm and term infants. Secreted peptides have been shown to have important biological functions. Neuropeptide Y, a 36-amino acid peptide secreted by the hypothalamus, was found to play key roles in neurodegenerative diseases including modulation of neurogenesis, food intake, and thermogenesis [51]. Mao et al. showed that peptides derived from human beta-defensins are secreted by viable human cryopreserved amniotic membrane and exhibited direct antimicrobial effects against *P*. *aeruginosa* [52]. Further studies are needed to understand and explore the functions of secreted peptides from hUC-MSCs. In our study, we used *p* value to screen differentially expressed peptides between term and preterm groups. Although the false discovery rate (FDR) can detect differentially expressed peptides more accurately than *p* value, we would get very few peptides by FDR. Additionally, in order to identify more differentially expressed peptides, some studies also applied the *p* value to the analysis of peptides [53, 54]. Thus, in some condition, it is also appropriate to use the *p* value.

Subcellular location analysis of precursor proteins can help to better understand the source and potential functions of peptides. As shown in Fig. 3d, most precursor proteins were annotated as being part of organelles or membranes (56%). Notably, a small fraction of peptides were annotated as being derived from proteins located in the extracellular region, termed secreted proteins from hUC-MSCs. Classically, a large proportion of peptides (such as peptide neurotransmitters) are generated by the proteolysis of macromolecular proteins followed by release into the space outside of cells [55]. However, some other peptides containing one or more cleavage sites do not derive from endosomal processing [56]. These peptides are the N- or C-terminal peptides of their precursor proteins, rather than internal fragments [57]. In addition, the identification in our study of several peptides arising from secreted proteins raised the possibility that some bioactive peptides may be produced by enzymatic hydrolysis of extracellular proteins. These observations provide us with more methods to evaluate the potential functions of differentially expressed peptides in our study; additional studies are needed to ascertain whether these peptides are actually secreted from hUC-MSCs and have biological activities. From the results of IPA analysis, we found that a series of precursor proteins were involved in networks of developmental disorders, inflammatory responses, and organismal injury and abnormalities associated with premature infant diseases and most of these precursor proteins participated in the course of premature infant diseases according to the Open Targets Platform database. These observations raised the possibility that the peptides secreted by hUC-MSCs may have beneficial effects with a similar role of precursor proteins in neonatal respiratory diseases, which are worthy of in-depth functional studies.

As the functions of secretory peptides derived from hUC-MSCs were unclear, we assessed whether the peptides were located in the functional domains of their precursor proteins to analyze their functionality. Using the UniProt database, we discovered that 25 peptides were situated in the functional domains of their corresponding precursors. Peptides derived from Titin (^8990^ESDSG^8994^, ^26941^EGNKDD^26946^, ^6684^SSRLECKI^6691^, and ^19103^VVHAGGVIRIIAYV^19116^) and one peptide derived from striated muscle preferentially expressed protein kinase (^2669^SCTVAVARVPGKLAPPEVPQ^2688^) were located in Ig-like domains. The Ig-like domain was initially characterized as a structure composed of two sheets of antiparallel β strands [58]. Okano et al. reported that the Ig-like domain contributed to the maintenance of the structure, activity, and stability of metagenome-derived glycoside hydrolase family 9 endoglucanase [59]. Interestingly, the Ig-like domain made endoglucanase Cel9A from *Alicyclobacillus acidocaldarius* dependent on calcium [60]. Additionally, Ig-like domains have been shown to play various roles in functions [61, 62]. We also found that two peptides derived from MUC19 (^961^DDFMSSQN^968^ and ^1382^QNGIIVI^1388^) were located in the type D von Willebrand factor (VWFD) domain. This domain plays a substantial role in reducing soluble VWF binding to platelet GpIba and regulates platelet activation and adhesion [63, 64]. It also participates in fertilization, as it binds to sperm proteases [65]. Like the Ig-like domain, the VWFD domain is partially responsible for biological processes and functions. The above motif analysis may provide a new perspective for clarifying the possible roles of these newly identified peptides.

Based on the above results, we focused on exploring the potential role of differentially expressed peptides in developmental disorders and inflammatory responses. BPD is clearly one of the most common respiratory morbidity in preterm infants, and it may not simply be a consequence of lung immaturity [66]. And inflammation, one key contributor, often initiated by a pulmonary fetal inflammatory response, is aggravated by invasive or non-invasive mechanical ventilation and exposure to hyperoxia [67]. Given the large predominance of inflammation in the pathogenesis of BPD, we focused on the effect of peptides on inflammation response. A549 cells, a human lung carcinoma cell line, are often used for researches in pathological mechanism and treatment of BPD by A549 cells [68, 69]. And in our study, H_2_O_2_, known as a strong two-electron oxidant, could induce the production for TNF-α, IL-1β, and IL-6 in human lung epithelial cell line A549 in agreement with previous studies [70]. As known to all, primary human AECII are difficult to isolate and culture and also are mutable and unsuitable for experimental intervention, while A549 cells have similar biological characteristics to AECII cells [69], although embryonic alveolar epithelial cells are more in line with the purpose of the project and can be better used to evaluate the effects of peptides on the characteristics of lung development. However, this cell is difficult to obtain and easy to mutate [69]. Thus, we chose human lung epithelial cells A549 to explore the effects of differentially expressed peptides in vitro*.* Accordingly, to make a full understanding of these peptides, animal experiments will be carried out to support our researches in the following experiments.

We also observed that ^7118^TGAKIKLVGT^7127^ derived from MUC19 reduced the levels of TNF-α, IL-1β, and IL-6 in H_2_O_2_-treated A549 cells and the degree of decrease is related to the concentration of peptide as shown in Fig. 5. As a secreted mucin, MUC19 is released to the extracellular medium and has been identified in respiratory, digestive, and reproductive tracts [71]. It has been reported that MUC19 was differentially regulated after exposure to inflammatory cytokines [72]. And one recent study found that MUC19 peptides may enhance vaginal mucous immunity against infections [73]. These results indicated that ^7118^TGAKIKLVGT^7127^ derived from MUC19 may also play a role in protecting from inflammatory response stimulated by H_2_O_2_ in human lung epithelial cells. In addition, the other peptide ^508^AAAAGPANVH^517^ derived from SIX5 also reduced the expression levels of TNF-α, IL-1β, and IL-6 in H_2_O_2_-treated A549 cells only at the maximum concentration of 100 μM. It has been acknowledged that SIX5 was correlated with eye development [74] and myotonic dystrophy [75], which are related to embryonic and organismal development. Thus, we put forward one hypothesis that the hUC-MSCs from preterm infants may secrete protective substances such as peptides under stress. These findings indicated that two peptides may play an antiinflammatory role in the process of BPD. And previous studies have shown that the regulation of inflammation-related signal pathways such as p38 mitogen activated protein kinases (p38MAPK) signal pathways and nuclear factor-κB (NF-κB) signal pathway can reduce the level of pro-inflammatory cytokines, thus relieving the pulmonary inflammation of BPD [76, 77]. Combined with previous studies, we consider that the two peptides secreted from hUC-MSCs may decrease the production of pro-inflammatory cytokines through inflammation-related signal pathways and further researches need to be conducted to validate the possible pathway.

## Conclusion

As far as we know, no large-scale quantitative peptidomic analysis has been carried out on the secretory components of hUC-MSCs. Our study identified the differentially expressed peptides secreted by preterm and term hUC-MSCs using TMT-based LC-MS/MS technology. Furthermore, bioinformatics analysis of precursors predicted the possible functions of peptides that may be useful in the treatment of premature respiratory diseases in connection with inflammatory responses and developmental disorders. And we first investigated the antiinflammatory effect of the peptides ^7118^TGAKIKLVGT^7127^ derived from MUC19 and ^508^AAAAGPANVH^517^ derived from SIX5 on human lung epithelial cells. This study expands our knowledge of the hUC-MSC secretome and may provide insights into new therapy for premature respiratory diseases.

## Supplementary information

**Additional file 1: Figure S1.** Characterization of hUC-MSCs from preterm and term umbilical cords. (A) hUC-MSCs from preterm and term umbilical cords exhibited a fibroblast phenotype in culture. Adipogenesis capacity was confirmed by oil red O staining, and chondrocytes were evaluated by Alcian blue staining after differentiation. (B) Flow cytometry was used to detect the expression of positive markers (CD29, CD73, CD105) and negative markers (CD31, CD34 and HLA-DR) on hUC-MSCs.

**Additional file 2: Figure S2.** Identification of protein integrity of hUC-MSC CM from preterm and term infants by silver staining. The protein integrity of hUC-MSC CM was visualized by SDS-PAGE and silver staining (n=3 per group, P1-3 represent preterm infants and T1-3 term represent term infants).

**Additional file 3: Table S1.** Putative precursor proteins associated with diseases.

**Additional file 4: Table S2.** Precursor proteins involved in networks.

**Additional file 5: Table S3.** Differentially peptides located in functional domain based on Uniprot database.

**Additional file 6: Table S4.** Protein precursors and identified peptides related to respiratory system diseases.

## Data Availability

The datasets used and analyzed during the current study are available from the corresponding author on reasonable request.

## Abbreviations

UC-MSC: Umbilical cord mesenchymal stem cell
TMT: Tandem mass tag
CM: Conditioned medium
GO: Gene Ontology
IPA: Ingenuity pathway analysis
PVL: Periventricular leukomalacia
BPD: Bronchopulmonary dysplasia
NEC: Necrotizing enterocolitis
ROP: Retinopathy of prematurity
Ang-1: Angiopoietin-1
HO-1: Heme oxygenase-1
EPO: Erythropoietin
GLP-1: Glucagon-like peptide-1
CGRP: Calcitonin gene-related peptide
AECII: Alveolar epithelium type II cells
LC-MS/MS: Liquid chromatography-tandem mass spectrometry
DMEM/F-12: Dulbecco’s modified Eagle medium/nutrient mixture F-12
RT: Room temperature
SDS: Sodium dodecyl sulfate
MWCO: Molecular weight cutoff
MW: Molecular weight
PI: Isoelectric point
H_2_O_2_: Hydrogen peroxide
TNF-α: Tumor necrosis factor α
IL-1β: Interleukin 1β
IL-6: Interleukin 6
GA: Gestational ages
ADRA2A: Alpha-2A adrenergic receptor
AGO2: Protein argonaute-2
BIRC6: Baculoviral IAP repeat-containing protein 6
KALRN: Kalirin
KMT2C: Histone-lysine N-methyltransferase 2C
SLC2A4: Solute carrier family 2
SLC4A4: Electrogenic sodium bicarbonate cotransporter 1
CROCC: Rootletin
DENND2A: DENN domain-containing protein 2A
KLF14: Krueppel-like factor 14
LONP1: Lon protease homolog
ATN1: Atrophin-1
COL8A1: Collagen alpha-1 (VIII) chain
JAG2: Protein jagged-2
MUC19: Mucin-19
MPDZ: Multiple PDZ domain protein
SIX5: Homeobox protein SIX5
FZD2: Frizzled-2
EMT: Epithelial to mesenchymal transition
HCAECs: Human coronary artery endothelial cells
HUVECs: Human umbilical vein endothelial cells
VWFD: Type D von Willebrand factor
