# Supplementary figures and images for: Peptidome analysis of umbilical cord mesenchymal stem cell (hUC-MSC) conditioned medium from preterm and term infants
Source: Stem Cell Res Ther. 2020 Sep 23;11:414. doi: 10.1186/s13287-020-01931-0 (PMC7510303; doi:10.1186/s13287-020-01931-0)

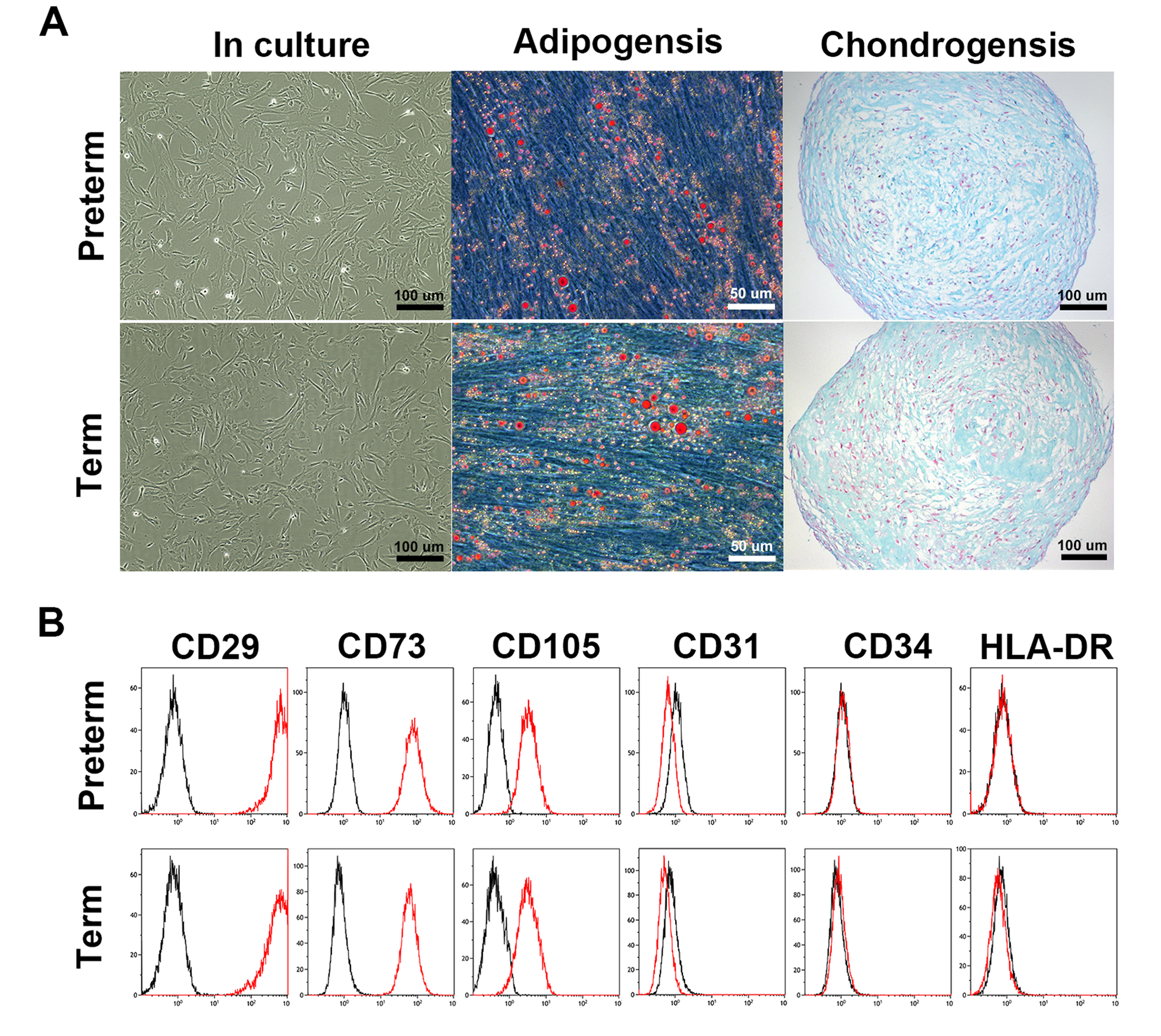

Supplement: Supplementary file 1 — Additional file 1: Figure S1. Characterization of hUC-MSCs from preterm and term umbilical cords. (A) hUC-MSCs from preterm and term umbilical cords exhibited a fibroblast phenotype in culture. Adipogenesis capacity was confirmed by oil red O staining, and chondrocytes were evaluated by Alcian blue staining after differentiation. (B) Flow cytometry was used to detect the expression of positive markers (CD29, CD73, CD105) and negative markers (CD31, CD34 and HLA-DR) on hUC-MSCs. [file 13287_2020_1931_MOESM1_ESM.tif]

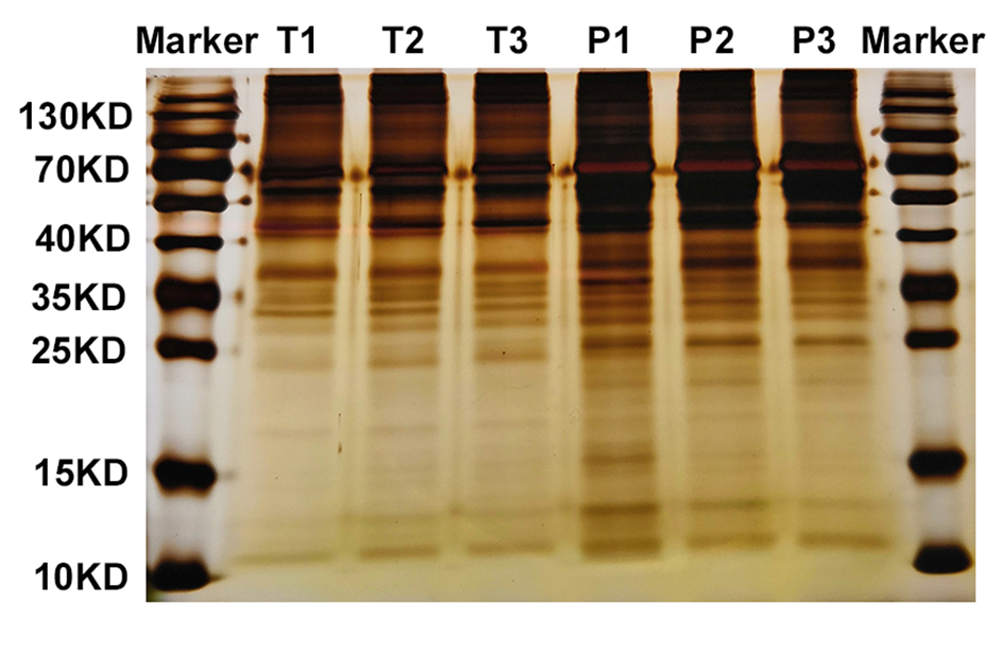

Supplement: Supplementary file 2 — Additional file 2: Figure S2. Identification of protein integrity of hUC-MSC CM from preterm and term infants by silver staining. The protein integrity of hUC-MSC CM was visualized by SDS-PAGE and silver staining (n=3 per group, P1-3 represent preterm infants and T1-3 term represent term infants). [file 13287_2020_1931_MOESM2_ESM.tif]
