# Supplementary material for: Peptidome analysis of umbilical cord mesenchymal stem cell (hUC-MSC) conditioned medium from preterm and term infants
Source: Stem Cell Res Ther. 2020 Sep 23;11:414. doi: 10.1186/s13287-020-01931-0 (PMC7510303; doi:10.1186/s13287-020-01931-0)
Supplement: Supplementary file 3 — Additional file 3: Table S1. Putative precursor proteins associated with diseases. [file 13287_2020_1931_MOESM3_ESM.docx]

| **Table S1 Putative precursor proteins associated with diseases** | | |
| --- | --- | --- |
| **Protein name** | **Underlying related diseases** | |
| **ADRA2A** | **Developmental Disorder** | **Inflammatory Response** |
| **AGO2** | **Developmental Disorder** | **Inflammatory Response** |
| **BIRC6** | **Developmental Disorder** | **-** |
| **KALRN** | **Developmental Disorder** | **-** |
| **KMT2C** | **Developmental Disorder** | **Inflammatory Response** |
| **SLC2A4** | **Developmental Disorder** | **Inflammatory Response** |
| **SLC4A4** | **Developmental Disorder** | **Inflammatory Response** |
| **STIM1** | **Developmental Disorder** | **Inflammatory Response** |
| **SYNE1** | **Developmental Disorder** | **-** |
| **THRA** | **Developmental Disorder** | **-** |
| **TSC2** | **Developmental Disorder** | **-** |
| **TTN** | **Developmental Disorder** | **-** |
| **GRHL3** | **Developmental Disorder** | **-** |
| **JAG2** | **Developmental Disorder** | **-** |
| **MTR** | **Developmental Disorder** | **Inflammatory Response** |
| **MYO10** | **Developmental Disorder** | **Inflammatory Response** |
| **PGR** | **Developmental Disorder** | **-** |
| **SPHK2** | **Developmental Disorder** | **-** |
| **APC2** | **Developmental Disorder** | **-** |
| **SCN11A** | **Developmental Disorder** | **-** |
| **SCN8A** | **Developmental Disorder** | **-** |
| **BSN** | **-** | **Inflammatory Response** |
| **CROCC** | **-** | **Inflammatory Response** |
| **DUSP10** | **-** | **Inflammatory Response** |
| **EPAS1** | **-** | **Inflammatory Response** |
| **IL17RC** | **-** | **Inflammatory Response** |
| **INPP5D** | **-** | **Inflammatory Response** |
| **LRP1B** | **-** | **Inflammatory Response** |
| **NPC1L1** | **-** | **Inflammatory Response** |
| **PIGR** | **-** | **Inflammatory Response** |
