# Supplementary material for: Peptidome analysis of umbilical cord mesenchymal stem cell (hUC-MSC) conditioned medium from preterm and term infants
Source: Stem Cell Res Ther. 2020 Sep 23;11:414. doi: 10.1186/s13287-020-01931-0 (PMC7510303; doi:10.1186/s13287-020-01931-0)
Supplement: Supplementary file 4 — Additional file 4: Table S2. Precursor proteins involved in networks. [file 13287_2020_1931_MOESM4_ESM.docx]

| **Table S2 Precursor proteins involved in networks** | |
| --- | --- |
| **Cellular Development, Embryonic Development and Organismal Development** | **Organismal Injury and Abnormalities** |
| **AGO2** | **ATN1** |
| **CDHR1** | **BAHCC1** |
| **CROCC** | **COL8A1** |
| **DENND2A** | **DUSP10** |
| **FGD5** | **GCN1** |
| **HECTD4** | **JAG2** |
| **JAG2** | **KALRN** |
| **KLF14** | **KMT2C** |
| **LONP1** | **MATR3** |
| **LRFN4** | **MMRN1** |
| **LRP1B** | **MUC19** |
| **MYO10** | **PDE4DIP** |
| **SKOR2** | **PIGR** |
| **SLC26A10** | **RNF123** |
| **TTC34** | **SCN11A** |
| **-** | **SCN8A** |
| **-** | **SNTB2** |
| **-** | **SPEG** |
| **-** | **TMOD2** |
| **-** | **TMPO** |
| **-** | **ZSWIM8** |
