# Supplementary material for: Peptidome analysis of umbilical cord mesenchymal stem cell (hUC-MSC) conditioned medium from preterm and term infants
Source: Stem Cell Res Ther. 2020 Sep 23;11:414. doi: 10.1186/s13287-020-01931-0 (PMC7510303; doi:10.1186/s13287-020-01931-0)
Supplement: Supplementary file 5 — Additional file 5: Table S3. Differentially peptides located in functional domain based on Uniprot database. [file 13287_2020_1931_MOESM5_ESM.docx]

| **Table S3 Differentially peptides located in functional domain based on Uniprot database** | | | | | | | |
| --- | --- | --- | --- | --- | --- | --- | --- |
| **Accession** | **Gene** | **Protein** | **Petide** | **Locations** | | **Domain** | **Description** |
|  |  |  |  | **Stard** | **End** |  |  |
| **Up-regualted peptides** | | | | | | | |
| **A0A0A0MTS7** | **TTN** | **Titin** | **ESDSG** | **8990** | **8994** | **8926 – 9014** | **Ig-like** |
| **Q9BYJ1-2** | **ALOXE3** | **Hydroperoxide isomerase ALOXE3** | **LNGRQQY** | **471** | **477** | **120 – 711** | **Lipoxygenase** |
| **Q15772-1** | **SPEG** | **Striated muscle preferentially expressed protein kinase** | **SCTVAVARVPGKLAPPEVPQ** | **2669** | **2688** | **2583 – 2673** | **Ig-like 9** |
| **Q7Z5P9-2** | **MUC19** | **Mucin-19** | **DDFMSSQN** | **961** | **968** | **815 – 1023** | **VWFD 2** |
| **E7EMZ9** | **TACC2** | **Transforming acidic coiled-coil-containing protein 2** | **RMSESPTPC** | **2520** | **2528** | **2620 – 2820** | **TACC_C** |
| **A0A0A0MTS7** | **TTN** | **Titin** | **LEDGG** | **21585** | **21589** | **21562 – 21658** | **Fibronectin type-III** |
| **Q9NRA0-3** | **SPHK2** | **Sphingosine kinase 2** | **EWDGIVTVSGDGLLHEVLN** | **201** | **219** | **178 – 325** | **DAGKc** |
| **Q8WXG9** | **ADGRV1** | **Adhesion G-protein coupled receptor V1** | **EAGLD** | **4310** | **4314** | **4255 – 4354** | **Calx-beta 29** |
| **Down-regulated peptides** | | | | | | | |
| **A0A0A0MTS7** | **TTN** | **Titin** | **KACDPVF** | **26480** | **26486** | **26389 – 26484** | **Fibronectin type-III** |
| **A0A0A0MTS7** | **TTN** | **Titin** | **IVASDVTKRLIKANLLANN** | **20618** | **20636** | **20572 – 20666** | **Fibronectin type-III** |
| **A0A0A0MTS7** | **TTN** | **Titin** | **EGNKDD** | **26941** | **26946** | **26880 – 26966** | **Ig-like** |
| **A0A0A0MTS7** | **TTN** | **Titin** | **DPPGKPVPLN** | **23926** | **23935** | **23927 – 24023** | **Fibronectin type-III** |
| **A0A140T8Y3** | **TNXB** | **Tenascin-X** | **HGRGRCEEGRCLCDPGYTGPTCATRMCPADCRGRGRCVQGVCLCHVGYGGEDCGQ** | **626** | **680** | **617 – 648** | **EGF-like** |
| **Table 3 (Continued)** | | | | | | | |
| **Accession** | **Gene** | **Protein** | **Petide** | **Locations** | | **Domain** | **Description** |
|  |  |  |  | **Stard** | **End** |  |  |
| **Q9HD67** | **MYO10** | **Unconventional myosin-X** | **KTSCVE** | **189** | **194** | **63 – 739** | **Myosin motor** |
| **I3L2R4** | **SLC2A4** | **Solute carrier family 2 (Facilitated glucose transporter), member 4, isoform CRA_b** | **IGAGVVNTVFTLVSVLLVERAGRRTLHLLGLA** | **327** | **358** | **27 – 461** | **MFS** |
| **A0A0A0MTS7** | **TTN** | **Titin** | **SSRLECKI** | **6684** | **6691** | **6667 – 6757** | **Ig-like** |
| **Q7Z5P9-2** | **MUC19** | **Mucin-19** | **QNGIIVI** | **1382** | **1388** | **1275 – 1484** | **VWFD 3** |
| **A0A0A0MTS7** | **TTN** | **Titin** | **VVHAGGVIRIIAYV** | **19103** | **19116** | **19090 – 19177** | **Ig-like** |
| **Q99707** | **MTR** | **Methionine synthase** | **KSARVMKKAVG** | **735** | **745** | **662 – 759** | **B12-binding N-terminal** |
| **Q8WXG9** | **ADGRV1** | **Adhesion G-protein coupled receptor V1** | **RFLQSIYLVPEEDHILIIPVVRGKDN** | **264** | **289** | **262 – 362** | **Calx-beta 3** |
| **O75970-3** | **MPDZ** | **Multiple PDZ domain protein** | **FISLLKT** | **1512** | **1518** | **1483 – 1564** | **PDZ 9** |
| **F8VZY0** | **MYBPC1** | **Myosin-binding protein C, slow-type** | **TDAKIFVRVKAVNAAGAS** | **815** | **832** | **735 – 846** | **Fibronectin type-III** |
| **Q9H3S7** | **PTPN23** | **Tyrosine-protein phosphatase non-receptor type 23** | **KLELLRQN** | **47** | **54** | **8 – 394** | **BRO1** |
| **Q9Y219** | **JAG2** | **Protein jagged-2** | **CGSDAGPGMPGTAASGVCGPHGRCVSQPGGN** | **589** | **619** | **574 – 634** | **EGF-like 10; atypical** |
