# Supplementary material for: Peptidome analysis of umbilical cord mesenchymal stem cell (hUC-MSC) conditioned medium from preterm and term infants
Source: Stem Cell Res Ther. 2020 Sep 23;11:414. doi: 10.1186/s13287-020-01931-0 (PMC7510303; doi:10.1186/s13287-020-01931-0)
Supplement: Supplementary file 6 — Additional file 6: Table S4. Protein precursors and identified peptides related to respiratory system diseases. [file 13287_2020_1931_MOESM6_ESM.docx]

| **Table S4 Protein precursors and identified peptides related to respiratory system diseases** | | | | |
| --- | --- | --- | --- | --- |
| **Accession** | **Gene** | **Protein Name** | **Peptide Numbers** | **Association score with respiratory system diseases** |
| **Q8TE73** | **DNAH5** | **Dynein heavy chain 5, axonemal** | **2** | **1.00** |
| **P08913** | **ADRA2A** | **Alpha-2A adrenergic receptor** | **1** | **1.00** |
| **Q9UI33-3** | **SCN11A** | **Sodium channel protein type 11 subunit alpha** | **1** | **1.00** |
| **P49815-3** | **TSC2** | **Tuberin** | **1** | **1.00** |
| **Q8NEZ4** | **KMT2C** | **Histone-lysine N-methyltransferase 2C** | **2** | **0.96** |
| **E7ERG8** | **LRP1B** | **Low-density lipoprotein receptor-related protein 1B** | **1** | **0.96** |
| **A0A140T8Y3** | **TNXB** | **Tenascin-X** | **1** | **0.89** |
| **Q99814** | **EPAS1** | **Endothelial PAS domain-containing protein 1** | **1** | **0.80** |
| **Q9UKV8** | **AGO2** | **Protein argonaute-2** | **1** | **0.77** |
| **Q9Y6W6** | **DUSP10** | **Dual specificity protein phosphatase 10** | **1** | **0.59** |
| **P06401-2** | **PGR** | **Progesterone receptor** | **1** | **0.59** |
| **Q8TEP8** | **CEP192** | **Centrosomal protein of 192 kDa** | **1** | **0.58** |
| **Q8WXG9** | **ADGRV1** | **Adhesion G-protein coupled receptor V1** | **2** | **0.57** |
| **A0A0A0MTS7** | **TTN** | **Titin** | **10** | **0.52** |
| **P36776** | **LONP1** | **Lon protease homolog, mitochondrial** | **2** | **0.51** |
| **A0A0C4DGG6** | **NPC1L1** | **NPC1-like intracellular cholesterol transporter 1** | **1** | **0.51** |
| **F8VZY0** | **MYBPC1** | **Myosin-binding protein C, slow-type** | **1** | **0.50** |
| **O14526-3** | **FCHO1** | **F-BAR domain only protein 1** | **1** | **0.50** |
